# Integrative Approach for Rehabilitation of an 11-Year-Old Child With Bilateral Hemiplegia

**DOI:** 10.7759/cureus.47950

**Published:** 2023-10-30

**Authors:** Vaishnavi Hatwar, Snehal Samal, Pallavi Harjpal, Swadha P Udhoji

**Affiliations:** 1 Physiotherapy, Ravi Nair Physiotherapy College, Datta Meghe Institute of Medical Sciences, Wardha, IND; 2 Neurophysiotherapy, Ravi Nair Physiotherapy College, Datta Meghe Institute of Medical Sciences, Wardha, IND

**Keywords:** hemiplegia, rehabilitation, physical therapy, stroke, ischemic stroke

## Abstract

Over 80% of stroke patients suffer from the most frequent form, ischemic stroke. It is rare in the pediatric age group, with an estimated frequency of 1.6 per 100,000 per year. In this case report, we reviewed a case of an 11-year-old female child with bilateral hemiplegia. Motor impairments are defined as weakness or paralysis on the opposite side of the body from the lesion (hemiplegia or hemiparesis). Complications and impairments include difficulty swallowing or eating, communication difficulties (both receptive and expressive), emotional changes, loss of bladder or bowel control, muscle and nerve diseases, and language, speech, and memory problems. A patient-centered approach to rehabilitation interventions was given. The patient's functional ability was greatly enhanced due to the physiotherapy rehabilitation we used. Effective rehabilitation has taken advantage of the brain's capacity for repair and recovery. A patient-centered approach to rehabilitation interventions promotes healing and independence through restitution, compensation, and prevention. Task-oriented training using motor learning constructs, coupled with exercise science, forms the basis of the intervention. Every outcome measure that was used showed improvement in the patient.

## Introduction

A stroke or cerebral vascular accident (CVA) is an abrupt loss of brain function brought on by a disruption in the blood flow to the brain. A stroke may be hemorrhagic, ischemic, or both. The most typical kind of stroke is an ischemic one, accounting for more than 80% of stroke patients, and can be caused by thrombosis, embolism, or hypoperfusion. Blood leaks into or around the brain during a hemorrhagic stroke owing to blood vessel rupture [1]. In the pediatric age group, acute ischemic stroke is an uncommon occurrence with an estimated incidence of 1.6 per 100,000 per year [2]. Neurological impairments must last at least 24 hours to be categorized as a stroke. Motor deficits include weakness (hemiparesis) or paralysis (hemiplegia) on the side of the body opposite the injury. Residual neurological impairments last over three weeks and can lead to lifelong disability.

Ischemic strokes are caused by thrombus, embolism, or other factors that induce low systemic perfusion pressures. The resulting decrease in cerebral blood flow deprives the brain of oxygen and glucose, alters cellular metabolism, and causes tissue death and damage. Ischemic strokes result in cerebral edema, an accumulation of fluids within the brain that begins minutes after the injury and peaks three to four days later. The swelling progressively diminishes and, in most cases, resolves within 2 to 3 weeks. Cerebral edema, which develops in big infarcts affecting the middle cerebral artery (MCA) and the internal carotid artery (ICA), is the major cause of death in acute stroke [3].

The most common symptoms of the anterior cerebral artery (ACA) syndrome are contralateral hemiparesis and sensory loss, with lower extremity (LE) involvement being greater than upper extremity (UE) involvement because the somatotopic organization of the cortex includes the functional area for the LE. MCA syndrome is distinguished by contralateral spastic hemiparesis and sensory loss of the face, UE, and LE, with the face and UE being more affected than the LE. Occlusion of the ICA usually results in a large infarction in the MCA-supplied area of the brain. The MCA and ACA are both supplied by the ICA [3].

Swallowing or feeding issues, communication difficulties (both receptive and expressive), emotional changes, loss of bladder or bowel control, muscular and nerve disorders, and language, speech, and memory problems are examples of complications and impairments. Effective rehabilitation has taken advantage of the brain's capacity for repair and recovery. A patient-centered approach to rehabilitation interventions promotes healing and independence through restitution, compensation, and prevention. Task-oriented training using motor learning constructs, coupled with exercise science, forms the basis of the intervention.

## Case presentation

An 11-year-old female child with right-hand dominance who lived in a rural area came to the hospital with her father following a chief complaint of fever and right-side weakness with abnormal movements for five days. She had a history of a fall on her head while playing three months back. As narrated by the father, the patient was well before five days, then she had a high-grade fever, which was intermittent and relieved by medication. She also had a weakness with abnormal movements without loss of consciousness, drooling of saliva, or uprolling of the eyeball. Then, her parents took her to a private hospital where initial management was done and advised them to do some investigation. Following that, studies such as CT scans and MRIs were carried out. There, she was diagnosed with a stroke. They were referred to our tertiary care hospital for further treatment. She was admitted to the ICU for three days. Then, she was transferred to the ward. Other medical treatment was continued and was advised for the physiotherapy.

Clinical examination

**Figure 1:**
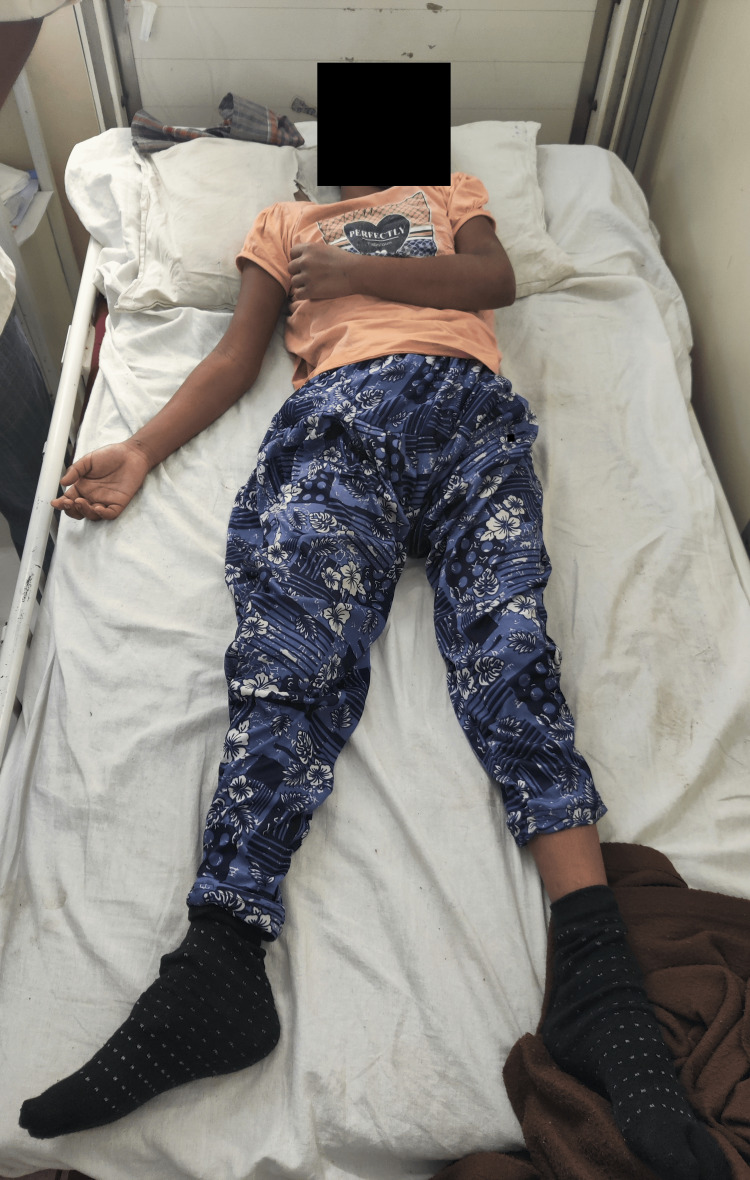
Image showing the attitude of the limb of the patient

The patient was examined supine on the bed with a pillow under her cervical spine for support. Vital signs were steady on general examination. She appeared alert and cooperative. The abduction of the shoulder and the forearm's supinated elbow and sideways extended wrist were observed to represent the right upper limb (UL) position. The left side exhibits internal rotation, adduction of the shoulder, and flexion of the wrist and elbows. Her hips were externally rotated, abducted, and extended in the bilateral lower limb (LL), with the ankle in plantar flexion and the knee in extension (Figure 1). The results of the evaluation showed that the higher mental functions score was 05/30 on the Mini-Mental Scale Examination (MMSE) and 05/30 on the Montreal Cognitive Assessment (MOCA). There was no damage to the cranial nerves. Upon sensory evaluation, bilateral superficial sensations over the UL, LL, and trunk remained unaltered. On motor assessment, muscle tone was increased for the upper limb for shoulder flexors, elbow flexors, wrist flexors, hip flexors, knee flexors, and ankle plantar flexors on the left lower limb and normal for the right LL (Tables 1, 2). Her left UL and LL were graded 1 on Voluntary Control Grading (VCG), while her right UL was graded 2. Muscle strength for the right LL is 3/5. The superficial reflex is depicted in Table 3, and Table 4 shows the grading of deep tendon reflexes.

**Table 1:** Muscle tone of upper and lower limbs

Tone	Right	Left
Upper limb (Shoulder flexors, elbow flexors, and wrist flexors)	Hypertonic	Hypertonic
Lower limb (Hip flexors, knee flexors, and ankle plantar flexors)	Normal	Hypertonic

**Table 2:** Muscle tone as per modified Ashworth scale (MAS)

Muscle Tone	Grading	
As per MAS	RIGHT	LEFT
Shoulder flexors	1	2
Elbow flexors	1	2
Wrist flexors	1	2
Hip flexors	Normal	1+
Knee flexors	Normal	1+
Ankle plantar flexors	Normal	1+

**Table 3:** Superficial reflexes

Reflex	Response	
Superficial	Right	Left
Plantar	Extensor	Flexor

**Table 4:** Deep tendon reflexes ++: Normal; +++: hyperreflexia

Reflexes	Grading	
Deep tendon	Right	left
Biceps jerk	+++	+++
Triceps jerk	+++	+++
Supinator jerk	+++	+++
Knee jerk	++	+++
Ankle jerk	++	+++

**Figure 2:**
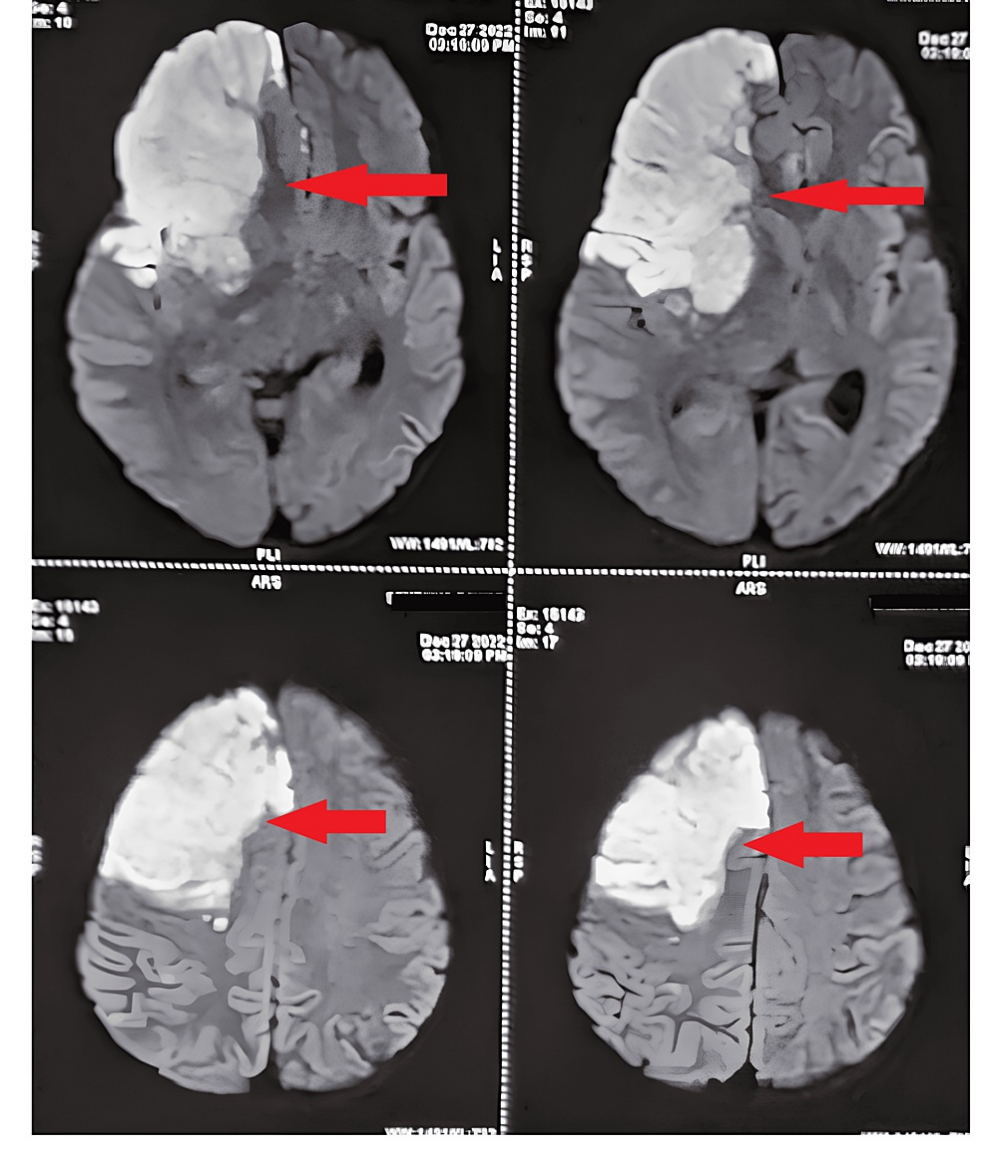
Magnetic resonance imaging (MRI) shows the infarct The red arrow shows the infarct in the brain.

On investigation, MRI of the patient reveals acute non-hemorrhagic right MCA territory infarct in the right frontal and temporal cortex and gangliocapsular region causing mass effect on the right lateral ventricle causing its effacement. Chronic lacunar infarct in the left centrum semiovale. Moderate luminal narrowing of the segment of left ICA and right MCA. Significant occlusion of bilateral anterior cerebral arteries. Figure 2 shows the MRI findings.

Diagnosis

The diagnosis of this patient is bilateral hemiplegia.

Therapeutic intervention

**Figure 3:**
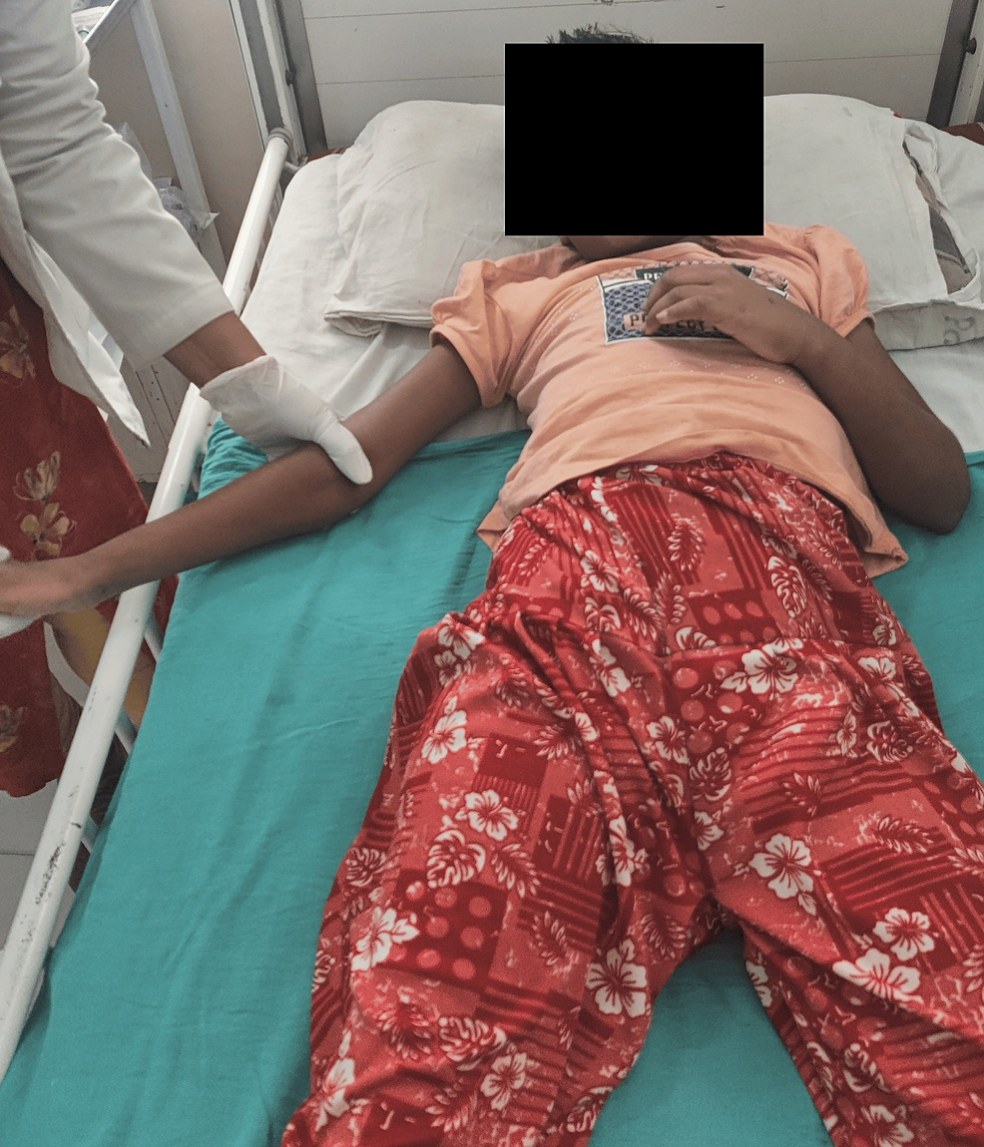
Proprioceptive neuromuscular facilitation (PNF) for right UL - D1 extension-abduction-internal rotation

**Figure 4:**
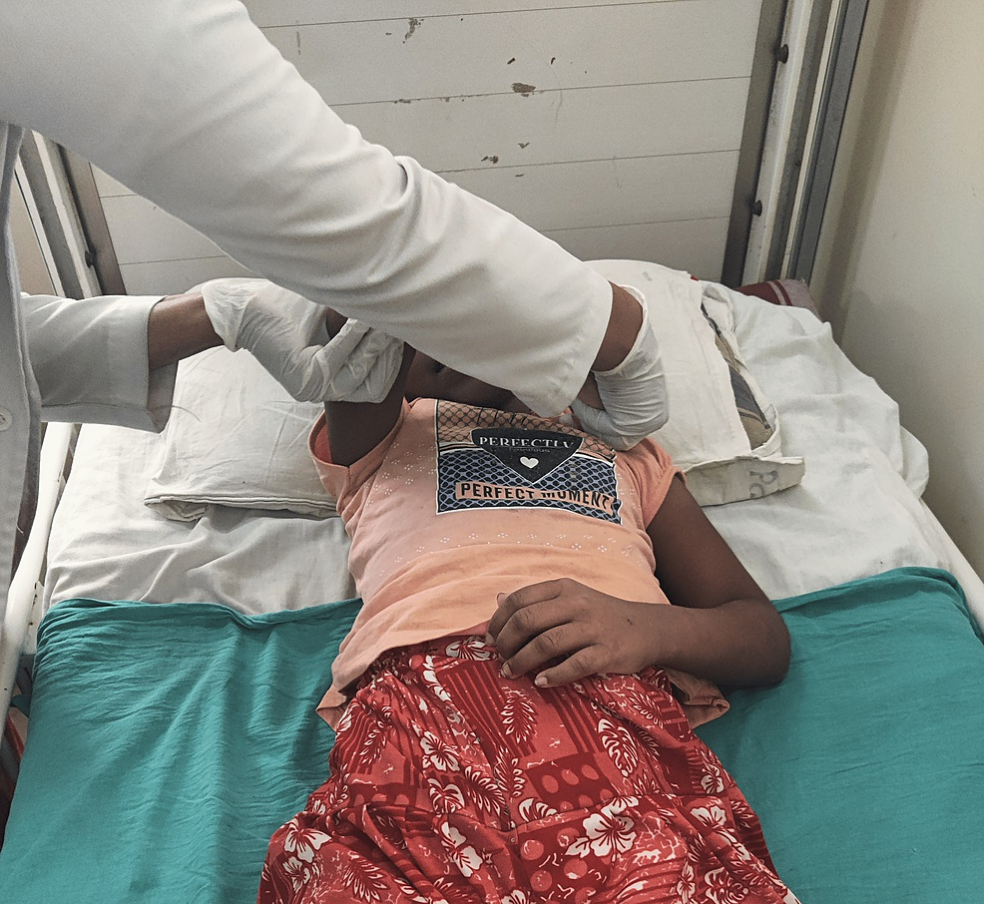
Proprioceptive neuromuscular facilitation (PNF) for right UL- flexion-adduction-external rotation

**Figure 5:**
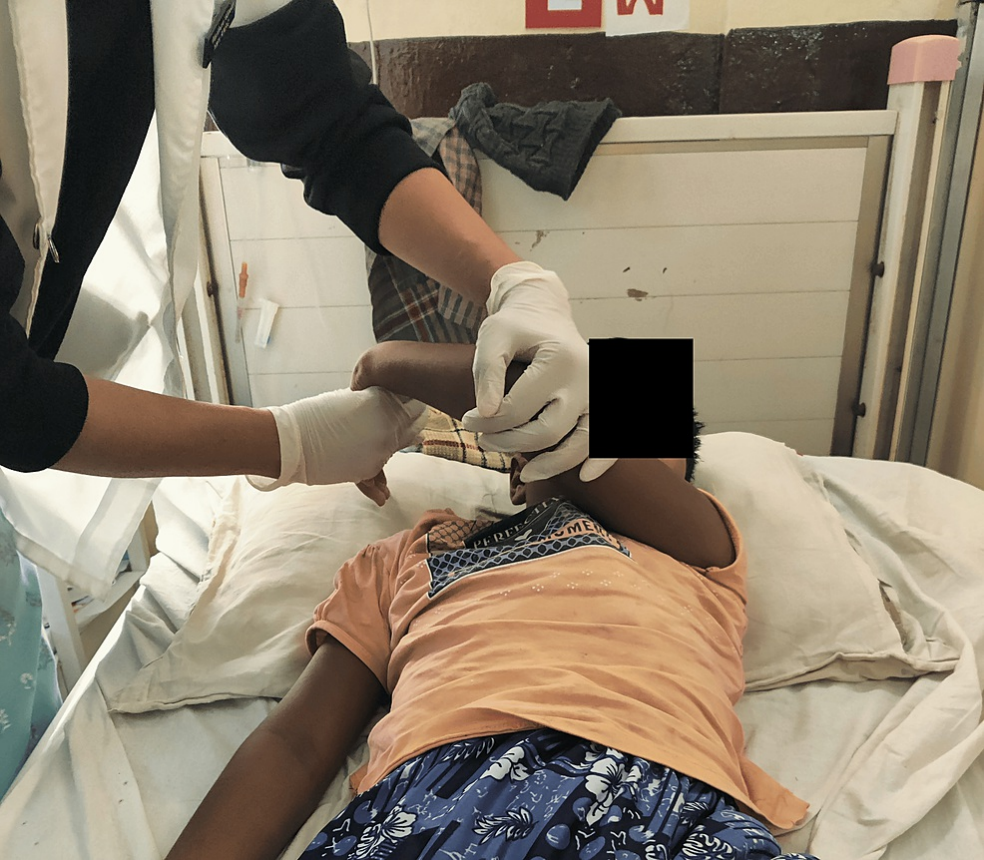
Proprioceptive neuromuscular facilitation (PNF) for left UL- flexion-adduction-external rotation

The patient was monitored in the ICU for a few days before being shifted to the ward. Physiotherapy sessions began as soon as the patient was moved to the ward. Vital signs were checked before and after the rehabilitation training and all the necessary precautions were taken. The intervention strategies are shown in Figures 3-5. The treatment protocol of the patient is given in Table 5.

**Table 5:** The treatment protocol of the patient UL: Upper limb; LL: lower limb

The problem faced by the patient	Rationale	Intervention and regime
Anxiety	To prevent anxiety and educate the patient and family about the condition	Patient and family counseling and education
Increased tone or spasticity	To reduce spasticity or inhibit abnormal tone	Rood's inhibitory technique (10 reps x 1 set)
Reduced bed mobility	To promote bed mobility and functional status. To avoid subsequent complications such as pressure sores and contractures.	Facilitate rolling - Using upper extremity momentum and crossing the ankle. Transitioning from supine to side lying, then to sitting. (10 repetitions x 1 set) And positioning every 2 hours was given.
Reduced range of motion and muscle strength	To improve the range and strength of UL and LL muscles. To improve coordination in functional activities.	Passive Range of Motion (PROM) exercises is for bilateral UL and LL. Active Assisted Range of Motion (AAROM) exercises for the right LL. Progression to the active range of motion (AROM). Proprioceptive Neuromuscular Facilitation (PNF) for UL D1 flexion-adduction-external rotation, D1 extension-abduction-internal rotation, D2 flexion-adduction-external rotation, D2 extension-adduction-internal rotation. (10 repetitions per set).
Tightness of muscle	To reduce the tightness of the muscle.	Stretching Tendo-Achilles (TA) and Hamstring muscle with 30-sec hold for 10 reps x 1 set.
Impaired trunk and hip control	To improve trunk and hip control	Pelvic bridging initiation then with 5 sec holds for 5 reps x 1 set progression to 10 sec holds for 10 reps x 1 set. Core strengthening exercises- Isometrics for core muscles (10 repetitions per set).
Unable to sit independently	To initiate sitting balance	Weight shifts side to side, scooting forward and backward (10 repetitions per set).
Reduced motor activity of upper extremity	To improve motor recovery. To improve the activity of more affected upper extremities.	Constraint Induced Movement Therapy (CIMT) The less affected arm hand was immobilized and repeated activities were performed with the more affected arm.
Impaired coordination of upper extremity	To improve the coordination of bilateral upper extremities.	Hand-Arm Bimanual Intensive Training (HABIT) includes activities such as catching a ball and holding things.
Reduced functional independence of the patient	For the awareness of the affected field of vision	Mirror therapy (10 reps x 1 set)
Reduced prehension activities	To facilitate gripping activities	Prehension exercises (10 reps x 1 set)
Difficulty in standing	To make the patient stand	Sit to stand (10 reps x 1 set).
Cognitive deficit - Problems with attention, memory, executive functioning, and information processing	To help a patient overcome all of these difficulties and to restore everyday functioning.	Cognitive therapy

Outcome measures

**Figure 6:**
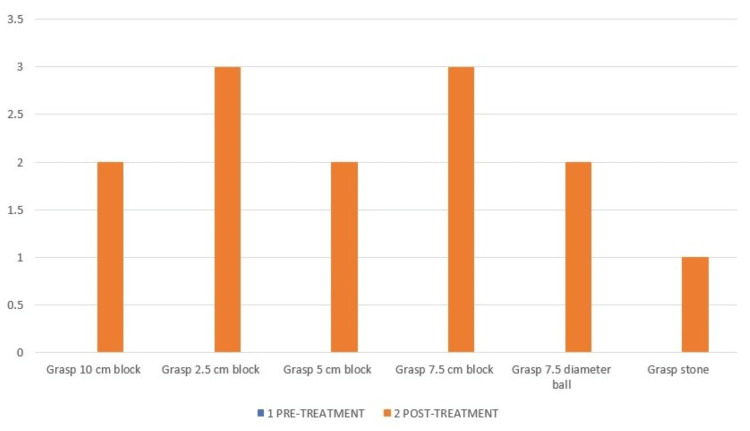
ARAT-Grasp component of pre- and post-treatment. The pre-treatment score in all the grasp components of ARAT was 0. ARAT: Action research arm test

**Figure 7:**
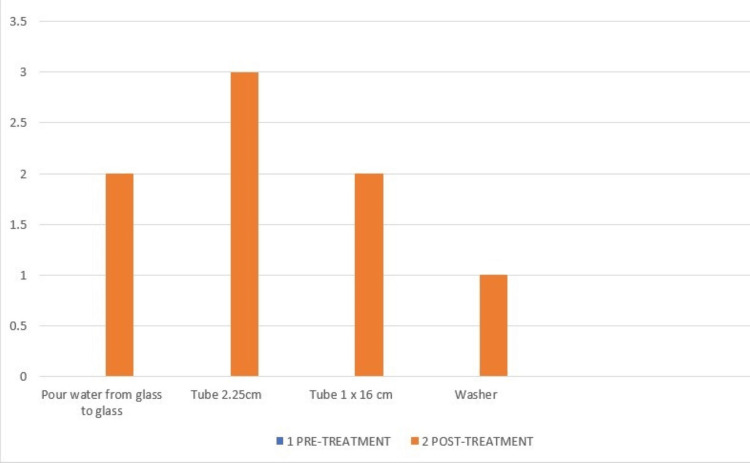
ARAT-Grip component of pre- and post-treatment. The pre-treatment score in all the grip components of ARAT was 0. ARAT: Action research arm test

**Figure 8:**
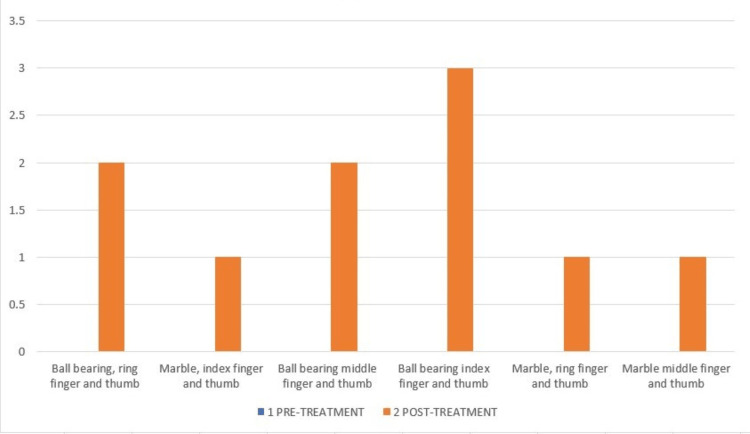
ARAT-Pinch component of pre- and post-treatment. The pre-treatment score in all the pinch components of ARAT was 0. ARAT: Action research arm test

**Figure 9:**
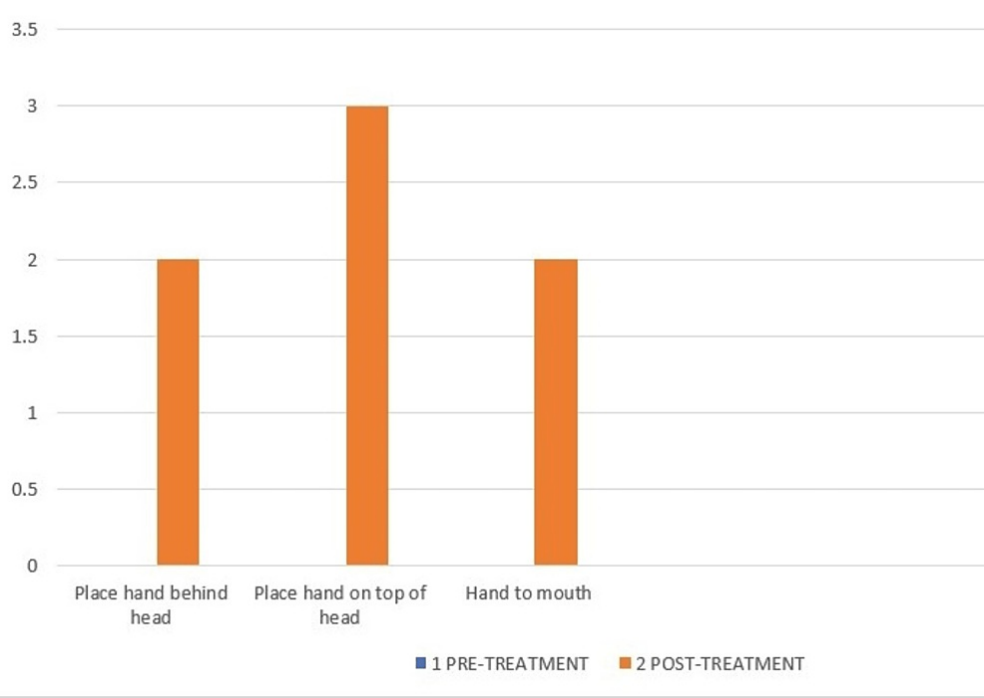
ARAT- Gross movements of pre- and post-treatment. The pre-treatment score in all the gross motor components of ARAT was 0.

The action research arm test (ARAT) consists of 19 items: grasp-six items, grip-four items, pinch-six items, and gross movements-three items, as shown in Figures 6-9.

**Figure 10:**
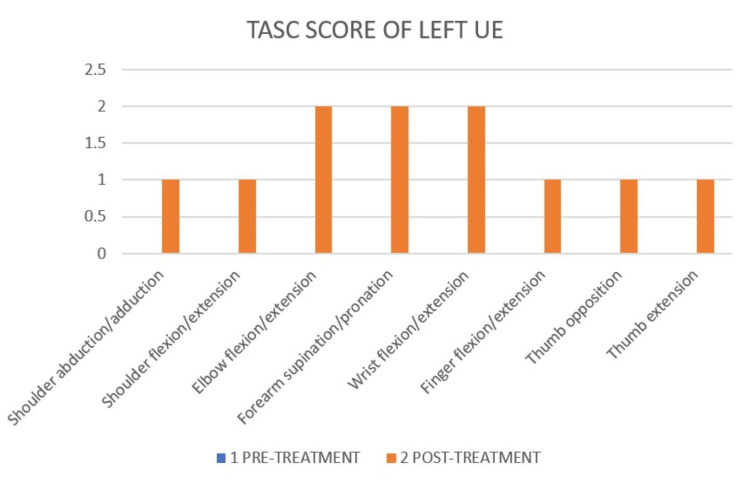
TASC score of left UE of pre- and post-treatment TASC: Test of Arm Selective Control; UE: upper extremity The pre-treatment TASC score of left UE was 0.

**Figure 11:**
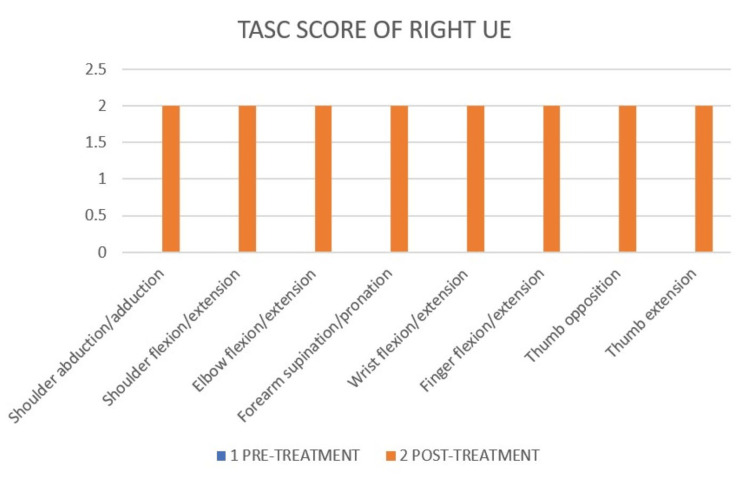
TASC score of right UE of pre- and post-treatment. TASC: Test of Arm Selective Control; UE: upper extremity The pre-treatment TASC score of right UE was 0.

Test of Arm Selective Control (TASC) differentiates and describes selective voluntary movement control in the upper limb, as shown in Figure 10 and Figure 11.

**Figure 12:**
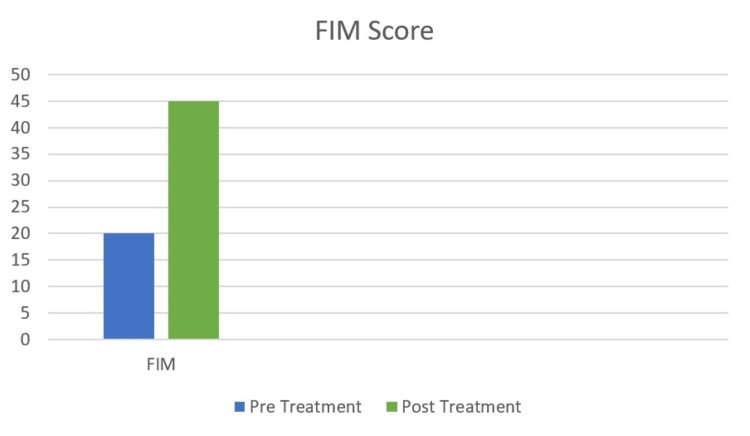
Functional independence measure of pre- and post-treatment. FIM: Functional independence measure

Functional independence measure (FIM) is an 18-item, 13-motor, and 5-cognitive task with a seven-level, ordinal scale. The score ranges from lowest 18 to highest 126, indicating the level of functioning. The pre-treatment score is 20, and the post-treatment score is 45, as shown in Figure 12.

**Figure 13:**
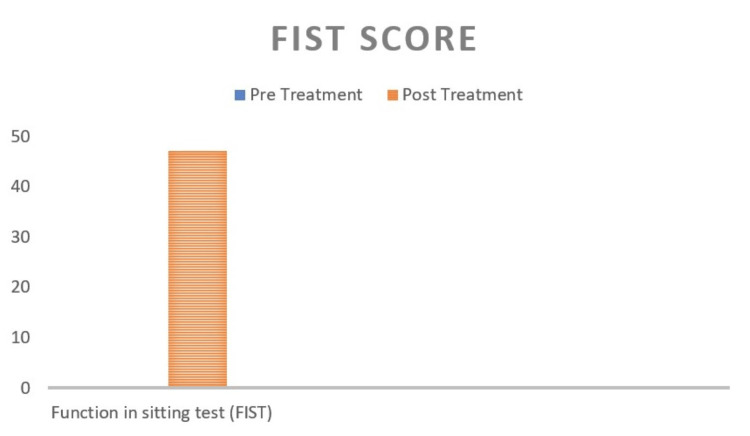
Function in sitting test (FIST) of pre- and post-treatment. The pre-treatment score of the FIST was 0.

The FIST, or function in sitting test, was created as a quick assessment of a patient's functional sitting balance in the wake of an acute stroke. The total score is 56. The pre-treatment score is 0, and the post-treatment score is 47, as shown in Figure 13.

## Discussion

Ischemic stroke in children is a rare event. Hemiplegia (paralysis) or hemiparesis (weakness) on the side of the body opposite the injury are the hallmarks of motor deficits. After three weeks, impairments may go away on their own as reversible ischemic neurological deficits. Residual neurological impairments last over three weeks and can lead to lifelong disability. Physical therapy promotes cortical restructuring and motor relearning, which aids in recovering motor function and functioning.

Rood's technique is based on established physiological facts that sensory stimulation produces the correct muscle response and is specifically created for people with motor control problems. Prolonged stretch, inhibitory tendon pressure, prolonged cold, and gradual rolling were given for inhibition. Electrical sensory input can significantly enhance motor function without affecting spasticity [4]. Mirror therapy (MT) demonstrated clear motor and sensory benefits. However, the level of improvement in sensory deficits and hemineglect is limited. MT is a successful and practical method of rehabilitating post-stroke survivors in the acute, subacute, and chronic phases of stroke [5]. A stroke might cause emotional disturbances and an immediate impairment in cognitive performance. After fewer than three months and six months of therapy, respectively, cognitive rehabilitation may help them with their attention deficits and activities of daily living (ADL) [6]. PNF approaches enhance functional movements by employing concentric, eccentric, and isometric contractions to facilitate, inhibit, strengthen, and relax muscle groups [7]. Hand-arm bimanual intensive training (HABIT) is a treatment that improves upper extremity function in children with cerebral palsy. In patients with acute stroke, HABIT markedly enhanced motor functional and neurophysiological outcomes [8]. Constraint-induced movement therapy (CIMT) is beneficial in both the subacute and chronic phases of stroke rehabilitation, and it is recommended in stroke clinical practice guidelines worldwide. CIMT may be a more effective treatment for hemineglect symptoms in acute stroke [9].

The patient received the aforementioned treatment because of her motor impairment, which included a reduced range of motion and muscle weakness in both her UL and LL, which limited her ability to perform ADLs, as well as because it is successful in reducing motor impairments and enhancing functional abilities in people with hemiplegia. The main objective of the physiotherapeutic intervention was to improve quality of life by increasing or restoring independence in ADLs. For this purpose, we focused on improving our strength and mobility. Furthermore, as UE became more involved, both gross and fine UE motor abilities were compromised, which were treated with neuro-physiotherapeutic therapies (ROM exercises, Rood's inhibitory methods, functional electrical stimulation, and prehension exercises), this resulted in the main goal of physiotherapeutic intervention being achieved, as evidenced by improvements in ARAT, TASC, and FIM scores.

## Conclusions

A patient-centered approach to rehabilitation interventions promotes recovery and independence through restitution, compensation, and prevention. The above-given protocol was effective in making the patient functional and independent. The given treatment made significant improvements in the patient's condition. This resulted in achieving the main goal of physiotherapeutic intervention, as evidenced by advances in ARAT, TASC, and FIM scores.
